# Association between physical activity, sedentary behavior, and fitness with health related quality of life in healthy children and adolescents: A protocol for a systematic review and meta-analysis

**DOI:** 10.1097/MD.0000000000006407

**Published:** 2017-03-24

**Authors:** Alberto Bermejo-Cantarero, Celia Álvarez-Bueno, Vicente Martinez-Vizcaino, Antonio García-Hermoso, Ana Isabel Torres-Costoso, Mairena Sánchez-López

**Affiliations:** a Health and Social Research Center, Universidad de Castilla-La Mancha, Cuenca; b Facultad de Ciencias de la Salud, Universidad Autónoma de Chile, Talca; c Laboratorio de Ciencias de la Actividad Física, el Deporte y la Salud, Facultad de Ciencias Médicas, Universidad de Santiago de Chile, USACH, Santiago, Chile; d School of Nursing and Physiotherapy, Universidad de Castilla-La Mancha, Toledo; e School of Education, Universidad de Castilla-La Mancha, Ciudad Real, Castilla-La Mancha, Spain.

**Keywords:** adolescents, fitness, health related quality of life, physical activity, schoolchildren, sedentary behavior

## Abstract

**Background:**

Health related quality of life (HRQoL) is a subjective, multidimensional and changing over time construct. When HRQoL is decreased, a child is less likely to be able to develop normally and mature into a healthy adult. Physical inactivity is a priority public health problem. Evidence suggests how even moderate levels of physical activity or high fitness levels are associated with benefits for the health in children and adolescents. The aims of this systematic review are to examine the evidence about the relationship between physical activity, sedentary behavior, and fitness with HRQoL, and estimate the effects of interventions that have tested the effectiveness of the increase of the physical activity, the improvement of the physical fitness or the avoidance of sedentary behaviors in HRQoL in healthy subjects aged under 18 years old.

**Methods:**

This systematic review and meta-analysis protocol was conducted following the preferred reporting items for systematic review and meta-analysis protocols (PRISMA-P) statement. To identify relevant studies, the following electronic databases will be searched: MEDLINE, EMBASE, Cochrane Database, Web of Science, and PEDro. Reference lists of relevant studies will be examined for links to potential related articles. The methodological quality of the observational included studies will be scored using a quality assessment checklist. For the intervention studies, the risk of bias will be estimated using The Cochrane Collaboration tool for assessing risk of bias. Reviewers will determine whether a meta-analysis is possible when data have been extracted. If it is, subgroup analyses will be carried out by age and socioeconomic status, and by the different dimensions of the HRQoL. If is not possible, a descriptive analysis will be conducted.

**Conclusion:**

To our knowledge, this systematic review and meta-analysis will be the first that synthesizes the existing results about the relationship between physical activity, sedentary behavior, physical fitness, and HRQoL, and the effect of physical activity interventions on HRQoL, in healthy subjects under 18 years old. This study will clarify this relationship and will provide evidence for decision-making. Limitations may include the quality of the selected studies and their characteristics. Only studies published in English and Spanish will be included.

**Systematic review registration:**

PROSPERO CRD42015025823.

## 1 Introduction

Quality of life (QoL) and, more specifically, health related quality of life (HRQoL), has been defined as the level of well-being derived from the evaluation that a person makes of diverse domains of his life, considering the impact these have on his health status.^[1]^ It is characterized as subjective, multidimensional, and changing over time.^[2]^ The QoL assessment incorporates at early ages the perception of physical, psychological, and social well-being according to evolutionary development and individual differences, within a specific cultural context, and considers the ability to fully participate in the activities and the physical, social, and psychosocial functions appropriate to their age.^[3]^ Children with poor HRQoL are less likely to develop normally and mature into a healthy adult.^[4]^

Many children and adolescents in developed countries lead sedentary lifestyles,^[5]^ with reduced active leisure activities, and increased reliance on sedentary lifestyles.^[6]^ Independent of physical activity levels, sedentary activities, especially those based on the use of electronic devices, are associated with an increased risk of obesity, and a reduction in physical condition, self-esteem, and prosocial behavior.^[7]^ In contrast, numerous studies have shown a positive association between physical activity and fitness levels with the physical, emotional, mental, and social health of children and adolescents.^[8,9]^

The relationship between physical activity, fitness, and HRQoL has been widely studied in both healthy and unhealthy adults.^[10–12]^ Similarly, the relationship between physical activity, HRQoL, and different pathologies such as diabetes,^[13,14]^ obesity,^[15–17]^ or cancer^[18,19]^ has also been largely studied in child and adolescent populations. Moreover, some studies have analyzed this relationship in healthy children and adolescents, suggesting a possible direct association between physical activity, fitness, and HRQoL,^[20–22]^ and an inverse relationship between sedentary behavior and HRQoL,^[23,24]^ but without conclusive results. On the other hand, it is also unclear how physical exercise interventions affect HRQoL in children or adolescents, since although some studies indicate that participation in physical activity programs may improve HRQoL or some of its dimensions,^[25,26]^ other studies have shown that HRQoL is unaffected by physical exercise interventions.^[27,28]^ There is a strong general belief that physical activity has many beneficial effects on HRQoL of youths, but to our knowledge, no systematic review and meta-analysis has synthesized the existing results.

## 2 Objectives

This systematic review and meta-analysis has two objectives to: (1) examine the relationship between physical activity, sedentary behavior, physical fitness, and HRQoL; and (2) estimate the effects of interventions that have tested the effectiveness of the increase of the physical activity (PA), the improvement of the physical fitness or the avoidance of sedentary behaviors in HRQoL in healthy subjects under 18 years old.

## 3 Methods

Our systematic review and meta-analysis protocol was developed following the preferred reporting items for systematic review and meta-analysis protocols (PRISMA-P) statement.^[29]^ The protocol for this review has been registered in the international prospective register of systematic reviews PROSPERO network (registration number: CRD42015025823).

### 3.1 Inclusion/exclusion criteria

#### 3.1.1 Types of studies

Interventional (randomized and non-randomized controlled trials and controlled pre-post studies) and observational studies (cross-sectional, longitudinal) written in English or Spanish will be eligible for inclusion.

#### 3.1.2 Types of participants

Inclusion criteria will be as follows: (1) children and adolescents from all countries aged <18 years old, and (2) has not been diagnosed with any pathology, including overweight and obesity. No sex, race, or socioeconomic status restriction will be applied.

#### 3.1.3 Types of interventions

Interventions that have tested the effectiveness of the increase of the PA, the improvement of the physical fitness, or the avoidance of sedentary behaviors in HRQoL targeted at the designated population will be included.

#### 3.1.4 Types of outcomes measures

Studies that report on HRQoL in healthy children or adolescents measured by validated questionnaires, both children self-reported and parent reported will be considered. Studies considering as outcome variables: “well-being,” “self-esteem,” “stress,” “psychological well-being,” or “mental health” will be excluded as these factors are related to QoL, but cannot be considered as specifically HRQoL-related. The variable physical activity (defined as any bodily movement produced by skeletal muscles that require energy expenditure) will be classified in terms of duration, frequency, intensity, and type. Data collection tools will include questionnaires, interviews, accelerometery, pedometer, heart rate monitors, or direct observation. Sports understood as the number of hours a week of practice of an organized sport. Sedentary behaviors could be classified in terms of screen time (TV, video games, and computer) and time studying, reading or doing homework, and could be measured by questionnaire or accelerometer. Cardiorespiratory fitness (understood as the capacity to perform prolonged exercise involving the cardiovascular and respiratory systems), and muscular fitness (understood as the capacity to generate force—muscular strength, to resist repeated contractions over time or to maintain a maximal voluntary contraction for a prolonged period of time—muscular endurance, and to carry out a maximal, dynamic contraction of a single muscle or muscle group in a short period of time—explosive strength, also called power.^[30]^ Both fitness components measured by laboratory test or by field tests that have shown validity and reliability in children and adolescents.

### 3.2 Information sources

#### 3.2.1 Electronic search

To identify relevant studies, the following electronic databases will be searched: MEDLINE (via PubMed), EMBASE, Cochrane Database (via The Cochrane Library), Web of Science, and PEDro.

After analyzing the key studies and considering expert recommendations, the following keywords were identified for conducting the search: physical activity, exercise, fitness, physical fitness, cardiorespiratory fitness, cardiovascular fitness, muscular fitness, muscular strength, muscular endurance, explosive strength, power, health related quality of life, well-being, positive health, psychological health, children, adolescent, young children, schoolchildren, sedentary behavior, screen time, screen based media use, effectiveness, trial, and intervention (Table 1).

**Table 1:**
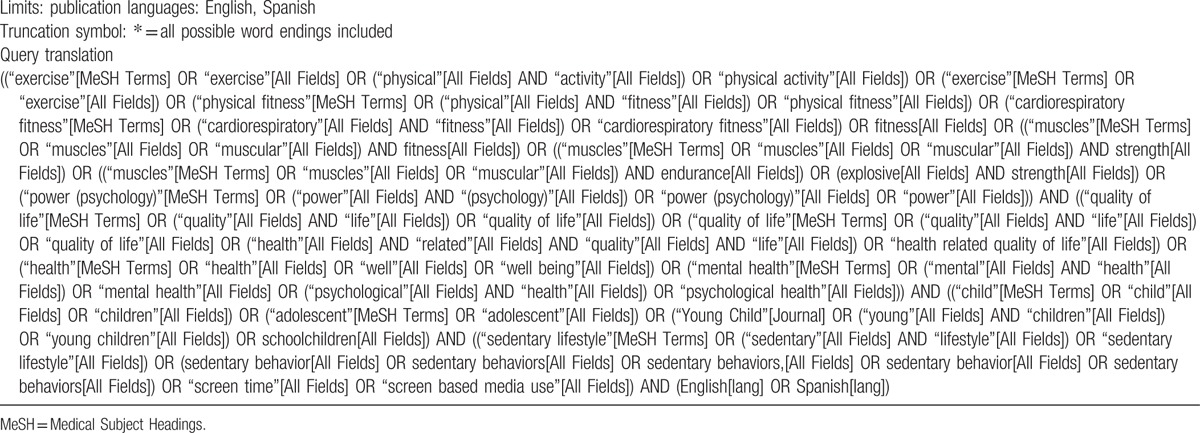
Sample search string for PubMed MEDLINE.

Two authors will screen titles and abstracts of the retrieved articles. Reference lists of the relevant studies will be further examined searching for other potential studies to include.

### 3.3 Data collection and analysis

#### 3.3.1 Data management

References will be imported into Endnote software (Thompson Reuters, CA). Duplicate articles will be removed and references assessed for eligibility will be alphabetically ordered, according to the first authors’ names.

#### 3.3.2 Selection process

Two authors (ABC and MSL) independently will screen the titles and abstracts of retrieved studies identified by the search strategy. Potentially eligible studies will be re-evaluated by reading the full text. In case of disagreement, the opinion of a third author (CAB) will be requested. Following PRISMA guidelines,^[31]^ a flow diagram will illustrate the study selection process.

#### 3.3.3 Data collection process

Data will be extracted in ad-hoc tables (Table 2). An author (ABC) will complete data extraction from selected studies (study design, year, country, number of participants, age, measuring instruments of physical activity, sedentary behavior, physical fitness, sport and HRQoL, relevant results and quality score). A second author (MSL) will check the accuracy and consistency of all inputs and will make the relevant clarifications when needed. A third author (CAB) will arbitrate unresolved disagreements regarding data extraction.

**Table 2:**
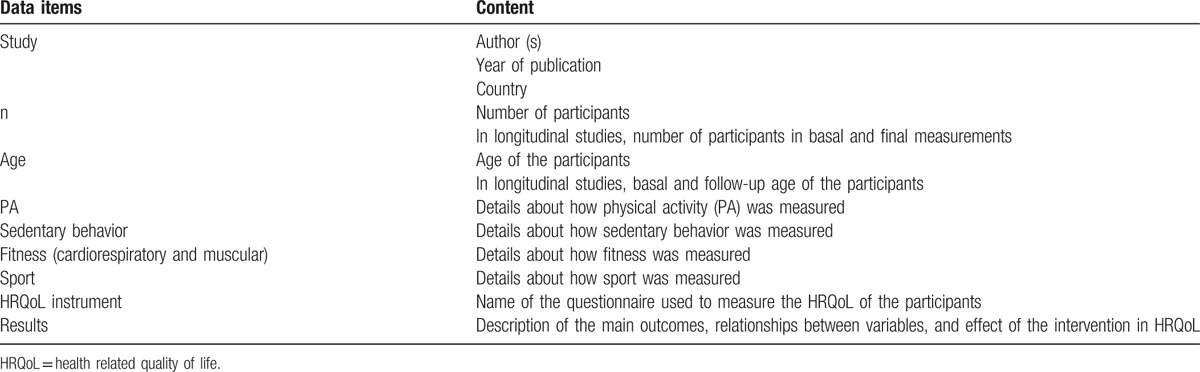
Data extraction variables.

#### 3.3.4 Quality assessment and risk of bias of included studies

Two authors (ABC and MSL) will independently assess the methodological quality of the studies. The results of this evaluation will be compared and the discrepancies will be discussed. A third author (CAB) will be consulted to resolve disagreements and Cohen *κ*^[32]^ will be used to assess agreement between reviewers when necessary.

The methodological quality of observational studies (cross-sectional and longitudinal) included will be scored using a quality assessment list, based on the list used by Ruiz et al.^[33]^ Through a consensual process, the modified tool has been developed and agreed. This tool evaluates 5 categories: study objectives, study population, exposure measurements and assessment of the outcome, and analysis and data presentation. The definitive checklist includes 10 items to assess the longitudinal studies and 8 items for the cross-sectional studies (Table 3). For each study, the items on the list will be rated as “positive” (+) or “negative” (−), depending whether or not they meet that point. When the information is unclear it will be qualified by a question mark (?). A total quality score will be calculated by counting the number of positive items (question marks will be counted as negative). Studies will be defined as high quality if they score positive for over 50% of the items (more than 5 points for longitudinal designs and more than 4 points for cross-sectional designs).^[34]^

**Table 3:**
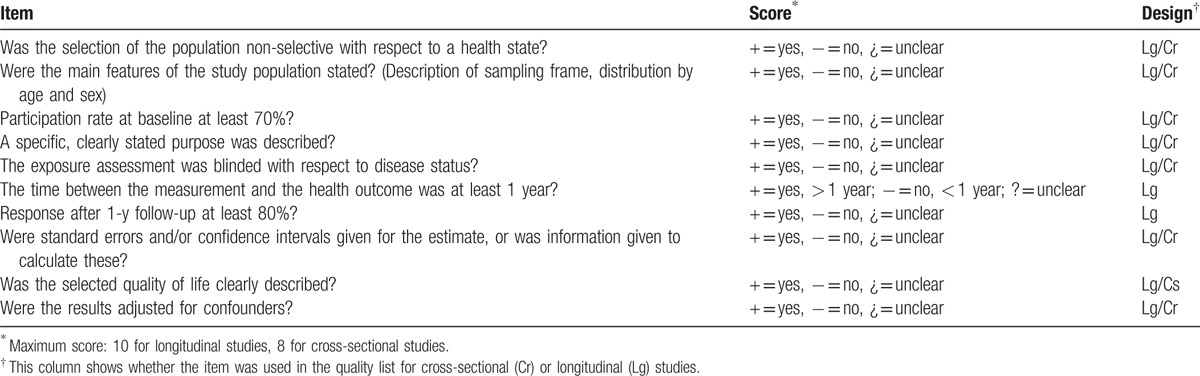
Quality assessment list of the cross-sectional and longitudinal studies.

For interventional studies, the risk of bias will be estimated using The Cochrane Collaboration tool for assessing risk of bias.^[35]^ This tool evaluates 7 specific domains: sequence generation, allocation concealment, blinding of participants and personnel, blinding of outcome assessment, incomplete outcome data, selective outcome reporting, and “other issues.” Each list item will be classified as “+” (low risk of bias), “−” (high risk of bias), and “?” (risk unclear), based on criteria for judging the risk of bias.^[35]^ The Revision Manager 5.3 software (The Nordic Cochrane Centre, The Cochrane Collaboration, Copenhague, 2014) will be used to introduce the different items and generate a “risk of bias” summary graph.

#### 3.3.5 Data synthesis

Ad hoc tables will be design for summarizing data of the included studies and show their key characteristics and any important questions related to the aim of this review. When data have been extracted, reviewers will determine whether a meta-analysis is possible. If it is, STATA 13 (StataCorp. 2013. Stata Statistical Software: Release 13. College Station, TX: StataCorp LP) software will be used to combine data, with 95% confidence intervals. Fixed-effect model analysis will be conducted if there is no evidence of heterogeneity, otherwise a random-effect model analysis will be performed. Heterogeneity will be assessed using the *I*^2^ statistic. Usually, *I*^2^ value is considered small if 0 ≤ *I*^2^ ≤ 25%, medium if 25% < *I*^2^ ≤ 50%, and large if *I*^2^ > 50%,^[36]^ also *P* values will be considered.

If there is substantial heterogeneity (in terms of common characteristic) among the studies and a meta-analysis is not possible, a descriptive analysis will be conducted.

#### 3.3.6 Sensitivity analysis

A sensitivity analyses will be conducted by excluding studies from the analysis one by one.

#### 3.3.7 Analysis of subgroups

Subgroup analyses will be performed by age, socioeconomic status, country groups (high-income vs. low-income) and population area (urban vs. rural). We also plan to carry out a subgroup analysis using the different dimensions of the HRQoL (physical, psychological, and social well-being).

## 4 Discussion

Previous systematic reviews have shown a consistent and positive association between physical activity level and HRQoL in the general adult population.^[10,11,37]^ To our knowledge, our proposed systematic review and meta-analysis will be the first to describe how PA, physical fitness, sport and sedentary behaviors are related to HRQoL in healthy children and adolescents, providing a detailed summary of the available evidence.

This review could be limited by the characteristics of the included studies and their quality. We will conduct and report our review using existing guidelines^[38,39]^ and will take into account potential risks of bias for each study.

Given the importance of the quality of life has for a good development in children and adolescents, a more detailed and comprehensive view on the effect of exercise and sedentary behaviors in the quality of life it is necessary. This protocol provides a clear and structured procedure for maximizing the extraction of relevant information and provides summarized information regarding the association between PA, fitness, sport and sedentary behaviors, and HRQoL in youth. The findings of this systematic review could be of interest for researchers, policy makers, and practitioners in the area of PA, education, and health care, providing knowledge as a basis for the development of effective action plans in the field of education and health.

### 4.1 Study status

Piloting of the study selection process.
